# Comparative analysis of morphological and molecular approaches integrated into the study of the dinoflagellate biodiversity within the recently deposited Black Sea sediments – benefits and drawbacks

**DOI:** 10.3897/BDJ.8.e55172

**Published:** 2020-08-18

**Authors:** Nina Dzhembekova, Fernando Rubino, Satoshi Nagai, Ivelina Zlateva, Nataliya Slabakova, Petya Ivanova, Violeta Slabakova, Snejana Moncheva

**Affiliations:** 1 Institute of Oceanology “Fridtjof Nansen”, Marine Biology and Ecology Department, Bulgarian Academy of Sciences, Varna, Bulgaria Institute of Oceanology “Fridtjof Nansen”, Marine Biology and Ecology Department, Bulgarian Academy of Sciences Varna Bulgaria; 2 Water Research Institute, Unit Talassografico “A. Cerruti”, National Research Council CNR-IRSA, Taranto, Italy Water Research Institute, Unit Talassografico “A. Cerruti”, National Research Council CNR-IRSA Taranto Italy; 3 National Research Institute of Fisheries Science, Research Center for Aquatic Genomics, Fisheries Research and Education Agency, Yokohama Kanagawa, Japan National Research Institute of Fisheries Science, Research Center for Aquatic Genomics, Fisheries Research and Education Agency Yokohama Kanagawa Japan; 4 Institute of Oceanology “Fridtjof Nansen”, Ocean Technologies Department, Bulgarian Academy of Sciences, Varna, Bulgaria Institute of Oceanology “Fridtjof Nansen”, Ocean Technologies Department, Bulgarian Academy of Sciences Varna Bulgaria

**Keywords:** Black Sea, phytoplankton, cyst, morphology, metabarcoding

## Abstract

One of the assets, assigned to the phytoplankton resting stages, is that of serving as the “memory” of the aquatic ecosystems and preserved biodiversity in the course of time. However, an accurate cyst identification proves to be a more difficult and extremely challenging process, even today. In order to gain a better taxonomic coverage of cyst assemblages in the Black Sea, an integrated approach of the classical morphological identification with metabarcoding methods (MySeq sequencing of V7-V9 regions of the 18S rDNA) was applied on thirteen surface sediment samples collected from different sites. A total number of 112 dinoflagellate taxa was detected at the species level and ascribed to 51 genera. In general, it is the molecular analysis that yields a higher number of taxa as compared to those obtained through the morphological taxonomy (66 taxa based on the DNA sequences versus 56 morphologically-identified taxa). Besides, it should be pointed out that the integrated dataset includes 14 potentially toxic dinoflagellate species. Discerned, subsequently, was a good dataset consistency for ten species, followed by some discrepancies as to a number of taxa, identified with one of the methods only, due to specific methodological biases. On the whole, it could be concluded that the combination of morphological and molecular methods is likely to increase the potential for a more reliable taxonomic assessment of phytoplankton diversity in marine sediments which, in turn, proves conclusively the utmost importance of the integrated approach.

## Introduction

Biodiversity of phytoplankton as key primary producers is of utmost importance for the state and activity of the marine ecosystems (Ptacnik et al. 2008) and the precise and accurate information about the species diversity is fundamental for the proper understanding of their functioning (McCann 2000, Strong et al. 2015). The production of dormant resting stages is a life-cycle trait common to many planktonic taxa (see Belmonte and Rubino 2019 for a review on this topic). Resting cysts accumulated in sediments will remain viable for years up to a century (Lundholm et al. 2011, Ribeiro et al. 2011), serving as potential seed banks and maintaining biodiversity over time (Kremp et al. 2015). At the same time, the occurrence of phytoplankton species in the water column is often discontinuous, with a vast number of taxa, some of them represented in a very small concentration, others growing in high abundance (sometimes forming blooms) for a very short period, followed by a marked decrease or even complete disappearance from the plankton community (Rubino et al. 1998, Moscatello et al. 2004). As a result, some species cannot be detected in routine monitoring programmes as viable cells, but are much more readily identified in the sediment at dormant stage only (Bravo et al. 2006, Rubino et al. 2010b, Salgado et al. 2011). Moreover, some of the resting cysts are produced by species causing harmful algal bloom (HAB) and play an important role in bloom initiation, representing a “dormant threat” for the ecosystem (Anderson et al. 2003, Garces 2004). Thus, the survey of cyst assemblages in recent sediments is a suitable tool, not only for studying phytoplankton biodiversity, but also for detecting harmful microalgae and species not previously reported in a given area (Rubino et al. 2010a).

Dinoflagellate cysts play a decisive role in the very functioning of marine coastal plankton, both from a biological and ecological point of view (Dale 1983) and information about their density and distribution add value to our knowledge of phytoplankton biodiversity (Bravo et al. 2006, Rubino et al. 2010a), improving our understanding of dinoflagellate population dynamics (Natsuike et al. 2013, Anderson et al. 2014) and predicting possible HAB events (Tang and Gobler 2012, Satta et al. 2013). Furthermore, having reliable data on the resting stage diversity is an essential prerequisite for accurate species identification. The existing species inventories of delineated dinoflagellate cysts are mostly based on morphological characteristics (Matsuoka and Fukuyo 2000) and it is precisely this classical approach that has been successfully applied in the Black Sea species inventory (Rubino et al. 2010b, Aydin et al. 2015, Mudie et al. 2017), regardless of the fact that there are some cases in which such an approach could lead to an ambiguous identification. In reality, however, many cyst morphotypes produced by Gonyaulacales, Gymnodiniales or Peridiniales species share the same “simple” subspherical shape with no evidence of any processes and are characteried by the following common distinctive features - the size, the presence of mucous or the outline of the archeopyle, when visible. This is precisely the reason why the recent molecular methods have appeared to be gaining in popularity when studying microbial diversity in sediment samples, unlocking their potential for more sensitive detection and exact taxonomical identification (Penna et al. 2010, Forster et al. 2016). Besides, the morphological analysis applied together with the next-generation sequencing (NGS) tool is likely to generate greater discrimination power giving a better insight into the resting stage communities in a given environment (Piredda et al. 2017, Jung et al. 2018). The classical morphological method proves to be particularly effective in estimating the abundance of different morphotypes, whereas the obtained environmental high-throughput sequence data are considered appropriate for a better assessment and re-evaluation of the taxon composition of resting propagules in the benthos.

The present study, taking a more integrated approach - i.e. combining next-generation sequencing of V7-9 hypervariable regions of the 18S rDNA with morphological observation, focuses exclusively on the dinoflagellate diversity and distribution in the Black Sea recent sediments as modern cyst assemblages. It is worth noting that the same NGS dataset has been previously used for assessment of overall phytoplankton composition in the Black Sea floor and which revealed a rich diversity of species not reported earlier in Black Sea sediments (Dzhembekova et al. 2018). Advanced in the study is a detailed comparative analysis of the dinoflagellate cyst diversity giving, thus, due prominence to the strengths and weaknesses of the two methods employed.

## Material and methods

### Sampling

**Figure 1.**
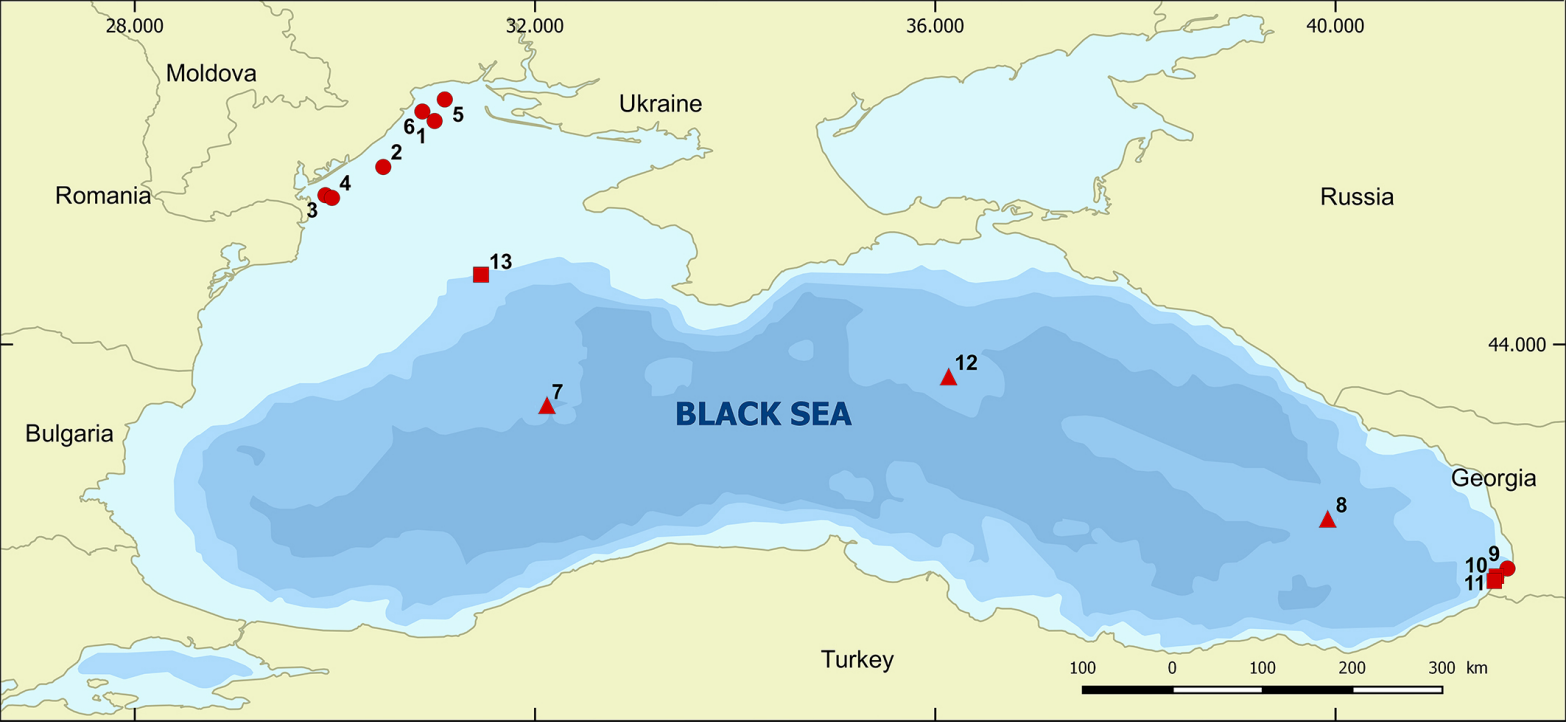
Map of sampling stations in the Black Sea (●coastal CO, ■shelf SH, ▲open sea OS)

Surface sediment samples were collected using a multicorer (the top 0–5 cm of the core) or Van-Veen Grab sampler, by a 10 x 10 cm frame at 13 stations located in different areas across the Black Sea during the period from May to June 2016 (Fig. 1, Table 1). All the samples were stored in a refrigerator at 8°C and kept in dark conditions in order to ensure only the survival of the resting stages in the sediments. The samples were well homogenised and split into two equal subsamples, each of which to be used for subsequent molecular and morphological analyses, respectively.

### Morphological identification

An aliquot of homogenied sediment (from 2.00 to 2.20 cm^3^) was selected from each subsample for the cyst analyses while another one (≈ 10 cm^3^) was concurrently oven-dried for one night at 70°C to calculate the water content. The wet aliquots were weighed and screened through a 10 µm mesh (Endecotts Limited steel sieves, ISO3310-1, London, England) using filtered natural (0.45 µm) seawater (Montresor et al. 2010). The material retained on to the sieve was then subjected to ultrasound for 1 min at low frequency and screened again through a sieve battery (200, 75, 20 and 10 µm mesh sizes). Two fine-grained fractions (10-20 µm and 20-75 µm), containing mainly protistan cysts, were obtained along with a > 75 µm fraction containing larger dinoflagellate cysts (e.g. *Lingulodinium* and *Polykrikos*) interspersed with zooplankton resting eggs. The material retained on to the 200 µm mesh was discarded. No chemicals were used to dissolve the sediment particles in order to preserve the calcareous cyst walls.

A quali-quantitative analysis for cysts taxonomic identification was conducted under an inverted microscope (Zeiss Axiovert 200M), equipped with a Leica MC17o HD digital camera at 320-400x magnification. Next determined was the number of both the full (i.e. with cytoplasmic content) and the empty (i.e. already germinated) cysts. As for the 10-20 µm and 20-75 µm fractions, at least 200 full cysts were counted in an attempt to obtain such values of abundance that are as homogeneous as possible and as a means to evaluate the rare species as well. From this perspective, a detailed and thorough analysis was performed on the > 75 µm fractions, while all the resting stage morphotypes were identified on the basis of published descriptions and the Modern Dinocyst Key Website (https://www.marum.de/en/Modern_Dinocyst_Key.html). The identification was performed at the species level whenever possible and, as a rule, the modern taxonomic appellation was applied. For those taxa whose active stages are not determined, the paleontological nomenclature was adopted. Quantitative data are reported as cysts x g^-1^ of dry sediment (cysts g^-1^).

### DNA extraction, PCR amplification, sequencing and bioinformatics

Total DNA was extracted from 0.5 g of surface sediment samples (in triplicate for each sampling location) using the ISOIL DNA extraction kit (NIPPON GENE, Tokyo, Japan). All DNA samples were used as templates for PCR amplification of the V7–9 hypervariable regions of the 18S-rRNA gene (amplicon length ~484 bp) using universal primers SSR-F1289-sn and F: TGGAGYGATHTGTCTGGTTDATTCCG; SSR-R1772-sn, R: TCACCTACGGAWACCTTGTTACG (modified from Tanabe et al. 2015). The massively parallel paired-end sequencing workflow was derived from the document 16S metagenomics sequencing library preparation: preparing 16S ribosomal gene amplicons for the Illumina MiSeq system distributed by Illumina (part no. 15044223 Rev. B). Two-step PCR for the construction of paired-end libraries and HTS on Illumina Miseq 300 PE platform (Illumina, USA) followed the protocols described in Dzhembekova et al. (2017). All the procedures and techniques, applicable to the treatment of the obtained sequences, selection and taxonomic identification of operational taxonomic units (OTUs), were administered according to the workflow described by Dzhembekova et al. (2017). When considering the taxonomic identification, a reference similarity threshold ≥ 98% was found appropriate.

DNA sequence dataset for this study can be found in the DDBJ Sequence Read Archive under access no. DRA009586.

### Statistical analyses

As similarity between morphological and molecular datasets is only possible for species identified by both methods, the two datasets were analysed under certain initial conditions: first, the datasets were aggregated and further referred to as coastal, shelf and open sea sampling sites (habitats) and, next, only species that were present at all sites were selected and viewed as “shared”. The shared species datasets comprised between 17 and 57% of the species numbers per station identified by the morphological method and between 12 and 32% of those specified by the molecular approach. The percentage fraction of each “shared” species in the total cyst abundance was calculated and expressed in numerical value per habitat.

Euclidean distances analysis was used for assessment of similarity between shared species datasets, by abundance. The data were normalised in the interval [0,1] and the resultant euclidean distances were further converted to similarity scores. The threshold similarity score value was set to 0.75 (*SimScore*_Treshold_ = 0.75) (Malone 2017).

## Results

### Morphological identification and enumeration of dinoflagellate cysts

From the total amount of 56 taxa, 51 were morphologically differentiated as full cysts and 33 as empty cysts, out of which 39 (almost 70%) were identified at the species level. Considerable spatial variability in cysts abundance has been observed, ranging between 269 (st. 9) and 6963 (st. 1) cysts g^-1^ for the full dinoflagellate cysts and between 66 (st. 9) and 5296 (st. 12) cysts g^-1^ for the empty dinocysts. On the whole, most species were unevenly spread. *Scrippsiella acuminata* (Ehrenberg) Kretschmann, Elbrächter, Zinssmeister, S.Soehner, Kirsch, Kusber & Gottschling, 2015 and *Scrippsiella* sp. dominated by frequency (found at all stations) and abundance (accounting for more than 70% of the total cyst abundance). Amongst the identified resting stages, five were assigned to potentially toxic dinoflagellates (*Alexandrium minutum* Halim, 1960, *Gonyaulax spinifera* (Claparède & Lachmann) Diesing, 1866, *Lingulodinium polyedra* (F.Stein) J.D.Dodge, 1989, *Polykrikos hartmannii* W.M.Zimmermann, 1930 and *Protoceratium reticulatum* (Claparède & Lachmann) Bütschli, 1885). Nonetheless, the cysts of these harmful microalgae were too sporadic and occasional to be found in great abundance with the exception of *L. polyedra*, identified at 11 out of 13 stations and measured in high densities (up to 1722 full cysts g^-1^).

The majority of cysts belong to the predominant Black Sea-specific phytoplankton species, except for those originally known as fossils only (*Calciodinellum operosum* Deflandre, 1947 †, *Calciperidinium asymmetricum* Versteegh, 1993 †, *Follisdinellum splendidum* Versteegh, 1993 †, *Melodomuncula berlinensis* Versteegh, 1993 †, *Posoniella tricarinelloides* (G.Versteegh) Streng, Banasová, D.Reháková & H.Willems).

### Dinoflagellate sediment diversity estimated on the basis of next-generation sequencing

The total number of dinoflagellate sequences in the dataset was 904,816 representing 36.4% of all sequences obtained by massively parallel sequencing (MPS). The sequences were clustered into 348 18S operational taxonomic units (OTUs) accounting for 15.5% of all the obtained OTUs. More than half of the dinoflagellate sequences (60.4%) could be assigned to references with high value of similarity varying from 100 to 98%. The number of OTUs that satisfied the taxonomic assignment criteria (> 0.980 BLAST top hit similarity) was 99 ranging by samples between 29 (st. 3) and 64 (st. 11). The number of sequences also fluctuated between samples with a minimum value of 18,871 (st. 8) and maximum approaching 87,693 (st. 7). More than 71% of OTUs (66 taxa) were determined at the genus level and 58% were identified at the species level (56 species) (Suppl. material 1). Only 7% of the OTUs, identified at the genus level, were assigned to reference sequences deposited in GenBank as sp. aside from the 6%, due to a similarity with two different species from the same genus. The remaining 29% of the OTUs in compliance with the assignment criteria could not be identified at a lower taxonomic rank due to a similarity with sequences deposited at a higher taxonomic level (6%) or through the same similarity with species from different genera (23%). The vast majority of OTUs were unevenly distributed amongst the stations, with a smaller number of species dominated by both sequence and sample numbers (e.g. *Biecheleria* sp., *Gymnodinium aureolum* (E.M.Hulburt) Gert Hansen, 2000). It is worth noting that, amongst the most abundant OTUs found at all the stations, some HAB species (*Gymnodinium catenatum* H.W.Graham, 1943 and *Karlodinium veneficum* (D.Ballantine) J.Larsen, 2000) were also preserved in the records. The selected OTUs, closely affiliated with potentially toxic dinoflagellates (*Alexandrium andersonii* Balech, 1990 and *Alexandrium pacificum* R.W.Litaker, 2014), were represented with just a few sequences and a single record, whereas the others, such as *Gonyaulax spinifera*, *Prorocentrum cordatum* (Ostenfeld) J.D.Dodge, 1975 and *Protoceratium reticulatum*, appeared more regularly with higher sequence numbers.

### Integrated morphological and molecular data

**Figure 2.**
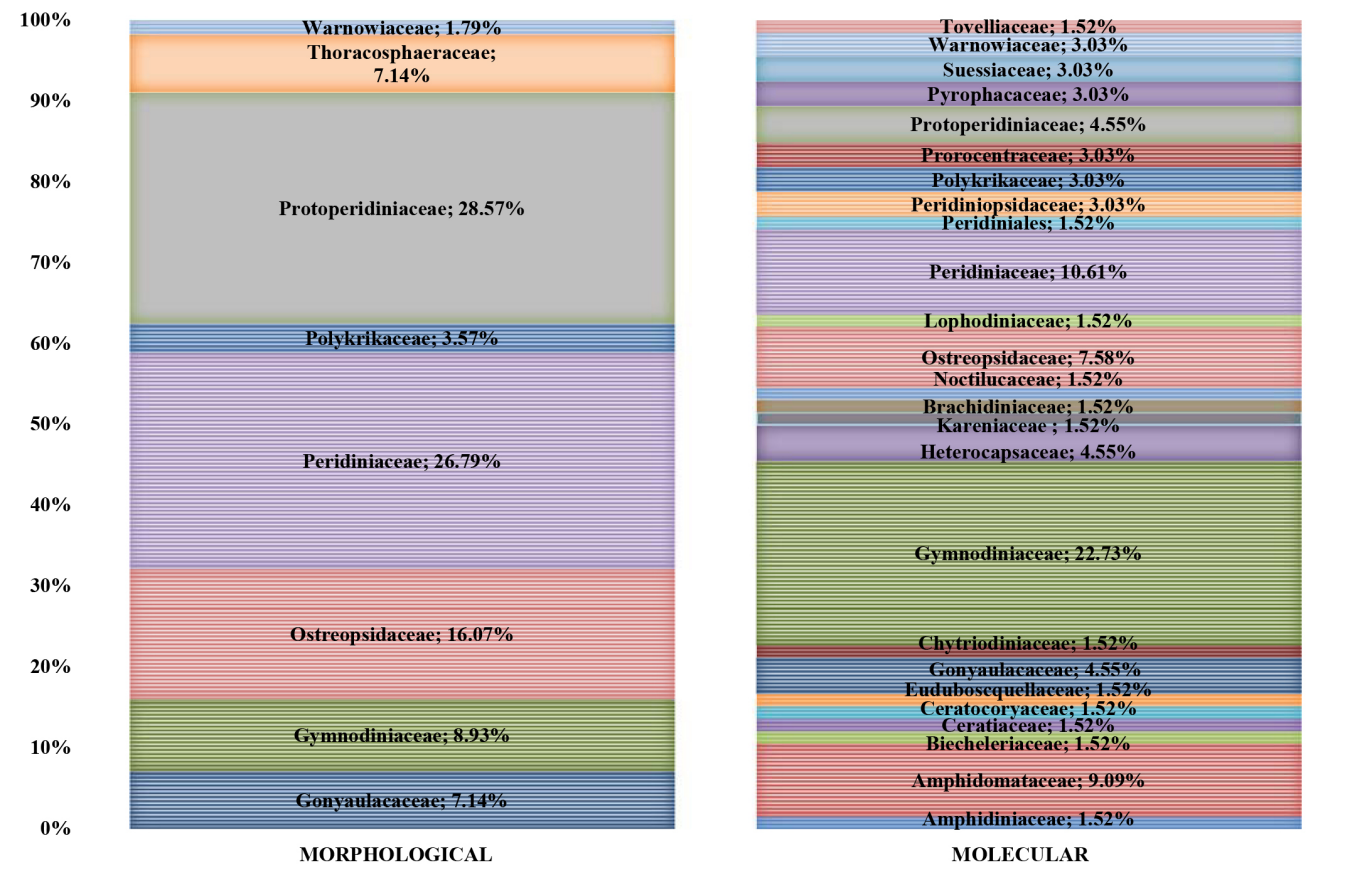
Family-level taxonomic compositions of dinoflagellates identified with the morphological and molecular approach.

The integrated morphological and molecular approach allowed for the detection of a total number of 26 dinoflagellate taxa at the family level, despite the inconsistent taxonomic composition between the morphologically- and metagenetically-derived datasets (Fig. 2). Some families were found only in the NGS dataset represented by a few species (OTUs), while the morphologically-identified fossil species in the family Thoracosphaeraceae escaped the molecular analysis probably due to the lack of a reference database. The highest taxonomic diversity was preserved in Peridiniaceae, followed by Protoperidiniaceae for morphologically-identified cysts while easily traced in Gymnodiniaceae and Peridiniaceae for molecular OTUs.

**Figure 3.**
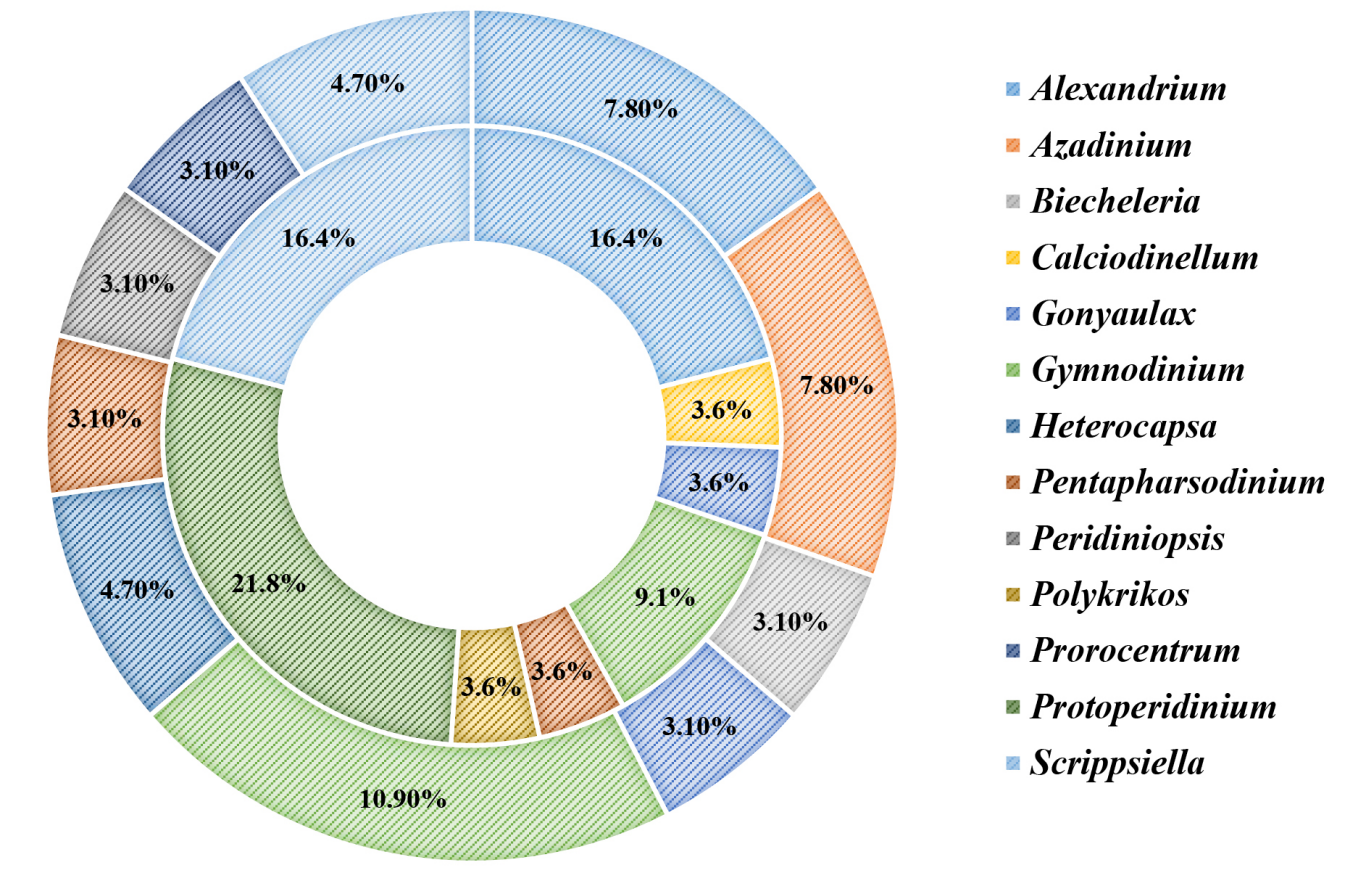
Species composition (number of taxa) percentage share by genera for the morphological dataset (inner ring) and the molecular dataset (outer ring). Only genera with percentage share ≥ 3% are presented on the chart (non-assigned taxa – below 3% represent 23.2% of morpohological and 48.8% of molecular datasets).

**Figure 4.**
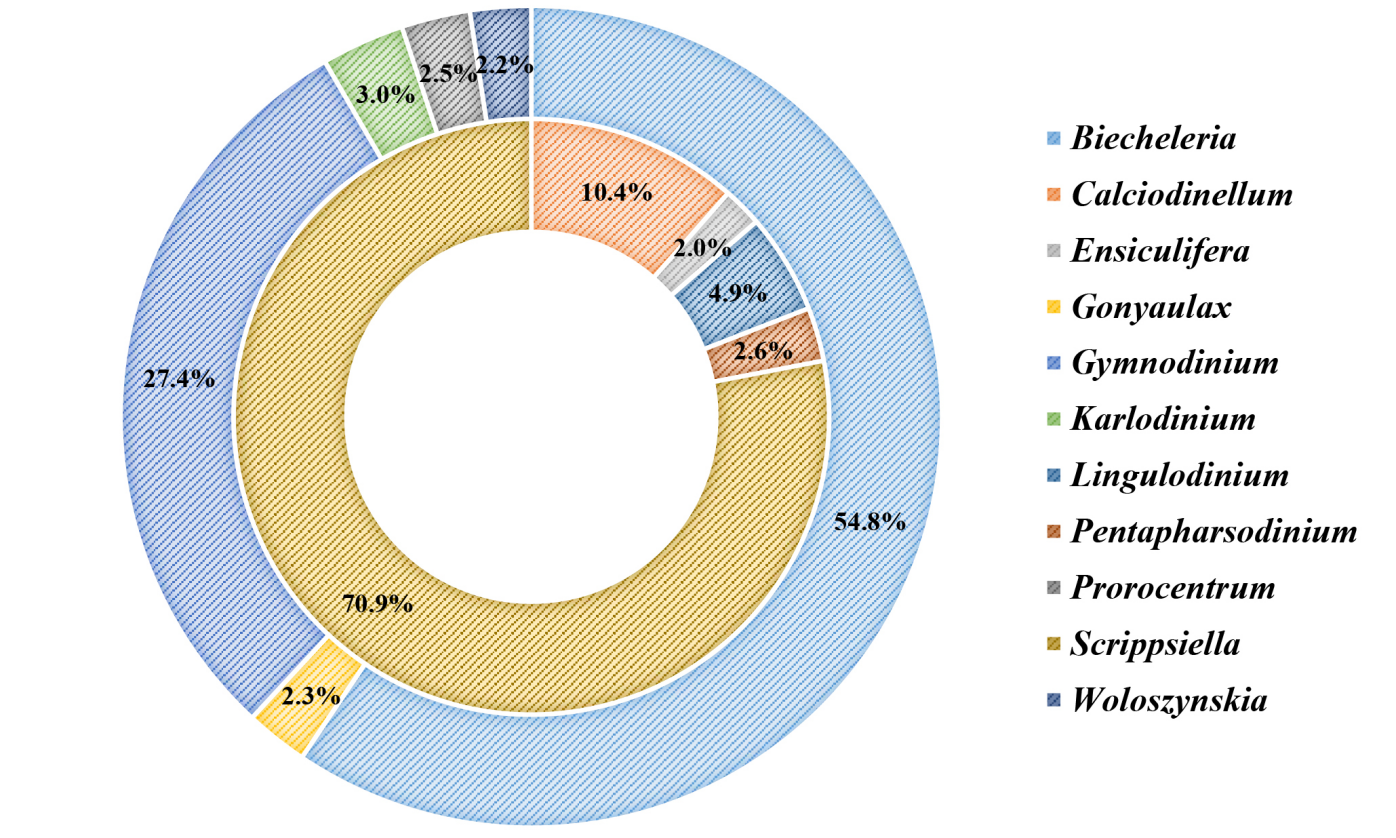
Abundance of taxa (cyst concentration and DNA sequence number) percentage share by genera for the morphological dataset (inner ring) and the molecular dataset (outer ring). Only genera with percentage share ≥ 3% are presented on the chart (non-assigned taxa – below 3% represent 11.11% of morphological and 17.83% of molecular datasets).

At the lower taxonomic level, 51 different genera were detected (21 retrieved by morphological/LM analyses and 41 by the molecular/NGS approach). Most of them were represented by a single species, each accounting for less than 3% of the total species number and only 13 yielding a higher proportion. *Alexandrium*, *Gonyaulax*, *Gymnodinium*, *Pentapharsodinium* and *Scrippsiella* were amongst the most diverse genera in both datasets (Fig. 3). By abundance, *Scrippsiella* dominated in the morphological dataset while *Biecheleria* and *Gymnodinium* were the taxa with the highest percentage in the molecular dataset (Fig. 4). Some of the dominant (most abundant and common) morphologically-identified genera, such as *Scrippsiella*, *Gymnodinium*, *Gonyaulax* and *Pentapharsodinium*, were clearly reflected in the NGS dataset. For the other taxa, there was not a good match between the two approaches. For example, *Alexandrium*, *Diplopsalis*, *Lingulodinium* and *Protoperidinium* were more frequent in the LM analyses, whereas *Polykrikos* and *Protoceratium* were detected in more samples via molecular analyses. Some dominant in NGS dataset genera (e.g. *Biecheleria*, *Biecheleriopsis*, *Heterocapsa*, *Karlodinium*, *Lepidodinium*, *Pelagodinium* and *Prorocentrum*) were overlooked by the classical method, just as others (e.g. *Ensiculifera*, *Kryptoperidinium*, *Nematodinium* and *Oblea*) that were subject to morphological identification only (Figs 3, 4).

Species-level taxonomic compositions revealed a total number of 112 dinoflagellates (56 - by the morphological approach versus 66 by the DNA sequences) with 85 species that could be clearly distinguished and the remaining ones identified as sp. (Suppl. material 1). The number of species detected by stations ranged between 11 (st. 10) and 32 (st. 4) for the morphologically-derived dataset and from 21 (st. 3) to 48 (st. 11) for the molecular dataset. In general, the total molecular classified OTU number was higher than the number of the morphologically-identified species in the samples. At one distinct station, there was a high discrepancy between the number of species identified through the morphological and molecular methods - the greatest difference was found at station 11, where 18 species were morphologically identified vs. 50 taxa detected via the molecular approach. From amongst the species readily distinguished, only 10 were shared between the two datasets (Table 2). Originally, the NGS method demonstrated higher sensitivity, detecting the species in more samples relative to LM, but the opposite was also noticed. Yet, in the final analysis, no correlation was found between the cysts abundance and the sequence reads for the shared species.

In total, 14 HAB dinoflagellate species were identified in the combined dataset, out of which five were accurately identified by the two methods. Apparently, the dominant species recorded with NGS (*Gymnodinium catenatum* and *Karlodinium veneficum*) were not present in the morphological results (*K. veneficum* is not known as a cyst producer). The potentially-toxic species detected by LM (*Alexandrium minutum*, *Gonyaulax spinifera*, *Lingulodinium polyedra*, *Polykrikos hartmannii* and *Protoceratium reticulatum*) were reflected in NGS data, but with a low degree of congruence.

**Figure 5.**
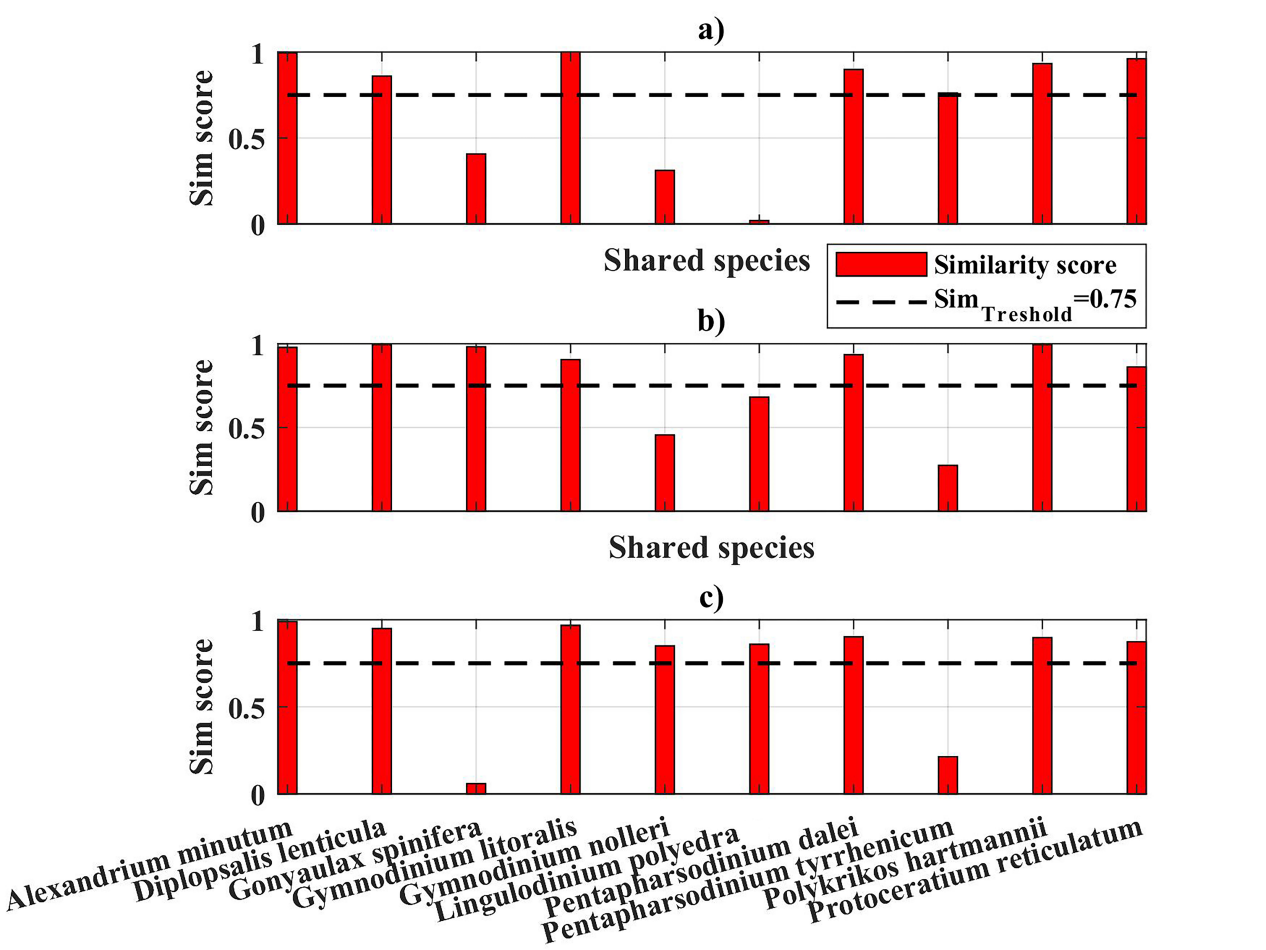
Similarity scores of shared species concentrations (identified by both morphological and molecular approaches) per habitat (resp.: a) coastal, b) shelf and c) open sea).

With regard to the shared species by habitats and in accordance with the initial conditions set above, the statistical analysis indicated that 7 out of 10 shared species (*Alexandrium minutum*, *Diplopsalis lenticula* Bergh, 1881, *Gymnodinium litoralis* A.ReÃ±é, 2011, *Pentapharsodinium dalei* Indelicato & Loeblich III, 1986, *Pentapharsodinium tyrrhenicum* (Balech) Montresor, Zingone & Marino, 1993, *Polykrikos hartmannii* and *Protoceratium reticulatum)* have a similarity score above the threshold (*SimScore*_Treshold_ = 0.75); for example, 70% overall similarity between datasets for the coastal habitat (Fig. 5). The same result was produced for the shelf habitat (e.g. 70% overall similarity between datasets), but the species with a similarity score above the threshold (*SimScore*_Treshold_ = 0.75), were slightly different *(Alexandrium minutum*, *Diplopsalis lenticula*, *Gonyaulax spinifera, Gymnodinium litoralis*, *Pentapharsodinium dalei*, *Polykrikos hartmannii* and *Protoceratium reticulatum*) (Fig. 5). In the open sea habitat, the comparative analysis showed that 8 out of 10 shared species, *Alexandrium minutum*, *Diplopsalis lenticula*, *Gymnodinium litoralis*, *Gymnodinium nolleri* M.Ellegaard & Ø.Moestrup, 1999, *Lingulodinium polyedra*, *Pentapharsodinium dalei*, *Polykrikos hartmannii* and *Protoceratium reticulatum*, have a similarity score above the threshold; for example, 80% overall similarity between datasets (Fig. 5). Evidently, the three habitats seemed to have 6 out of the 10 shared species in common: *Alexandrium minutum*, *Diplopsalis lenticula*, *Gymnodinium litoralis*, *Pentapharsodinium dalei*, *Polykrikos hartmannii* and *Protoceratium reticulatum*.

## Discussion

Principally, even nowadays, detailed and extensive research studies into the marine phytoplankton dormant stages in the Black Sea sediments remain scarce and limited compared to the intensive planktonic studies. The proposed study, therefore, was designed to give a sharper focus on species-level taxonomic composition, which yielded a total number of 56 morphologically-differentiated dinocyst taxa. Within them, 39 species were immediately identified, which is relatively high when compared with other morphology-based studies of recent Black Sea sediments (Rubino et al. 2010b, Aydin et al. 2015) and also represents quite a substantial proportion against those reported by Mudie et al. (2017), exceeding the number of 71, but at 185 datapoints. In addition, four different cyst types (data not shown) were identified for *Scrippsiella acuminata*, revealing much higher intraspecific morphological variability of these species over those reported previously (Rubino et al. 2017). The cyst concentration of *S. acuminata*, a typical bloom causing species in the Black Sea (Oguz 2008), as the most abundant cysts in the region, most likely is a good example of the existing benthic-pelagic coupling in the ecosystem processes. Nevertheless, there are some morphotypes of cysts associated with the genera *Gymnodinium*, *Alexandrium*, *Protoperidinium* and *Scrippsiella* for which it was not possible to be successfully identified to species level due to certain methodological constraints, for example, lack of clearly defined morphological features (Bolch et al. 1999, Bravo et al. 2006) or extremely low germination success. In case of difficult morphological identification, the general perception is that molecular methods could be the preferred option for dinocyst identification as it shows greater efficiency and higher resolution at species-level (Penna et al. 2010). In this study, metabarcoding data identified 66 dinoflagellate taxa with a high level of similarity with the reference database. Although the taxonomic coverage was fairly satisfactory, the identification power was contingent upon key issues of DNA methodology itself, such as insufficient resolution capacity of the barcode and the quality and completeness of the reference database.

Taking into consideration the advantages and drawbacks of the morphological and molecular methods and their appropriateness for biodiversity assessment, suggests that the two methods should be cross-checked if they are to ensure the accuracy of the dinocyst identification. To that effect, the integration and comparison of data derived by different approaches seem imperative, regardless of the subtle interpretation of the combined dataset. A critical issue in the comparative analyses is to use the same taxonomic concept in all attempted approaches as biodiversity can be measured at different taxonomic levels (Colwell 2009). Thus the detailed assessment of the comparability of the two methodologies, a comparison between the taxa lists derived from both NGS and morphological approaches revealed that basically, the DNA metabarcoding yielded higher taxonomic diversity than that derived by morphology. While all the families that were found by LM were recovered by NGS, metabarcoding data recovered the presence of 18 families that were not detected morphologically. Thus, it could be reasoned that the molecular approach was highly beneficial for detecting a higher number of taxa at the genus and species level, while both morphology and NGS seemed more efficient in revealing the degree of similarity between some of the dominant genera. Other taxa were further detected by either morphology or NGS methods exclusively. Our findings were in line with those indicated in other studies integrating molecular and morphological approaches, not only for resting cysts, but also for planktonic stages (e.g. Penna et al. 2010, Eiler et al. 2013, Zimmermann et al. 2015, Hirai et al. 2017, Jung et al. 2018, Perini et al. 2019). It should also be acknowledged that the discrepancy between LM and NGS could be attributed to a number of different methodological biases – from sampling to database biases (Matsuoka and Fukuyo 2000, Zimmermann et al. 2015, Danovaro et al. 2016, Dzhembekova et al. 2017, Harvey et al. 2017, Smith et al. 2017) (Table 3). The sampling biases can be primarily triggered by the limited amount of sediment subsample used for initial sample processing, both for DNA isolation and morphological analyses and most notably for rare species, i.e. present in very low densities. In addition, the natural heterogeneity of sediments should be considered as a possible cause of the wide disparity in the taxonomic composition and abundance. Amongst the limitations of the NGS method that may affect the reliability of the resting cysts detection, are the controversial issues concerning the DNA from the sedimented vegetative cells and those related to the extracellular DNA preserved in the sediment (Corinaldesi et al. 2011). Another factor that might explain the observed incongruities is the difficult morphological identification of some cysts; for example, a broad range of morphotypes assigned to *Alexandrium*, *Protoperidinium* and *Scrippsiella* could not be identified at the species level, as the lack of specific features has an adverse effect on the successful accuracy of the subsequent identification (Bravo et al. 2006, Matsuoka et al. 2006). The resolution of the primers applied was insufficient for some taxa with high intragenus and intergenus similarity. Thus, for instance, within *Biecheleria* (*Biecheleria brevisulcata* K.Takahashi & Iwataki, 2014 and *Biecheleria cincta* (Siano, Montresor & Zingone) Siano, 2012)), *Prorocentrum* (*Prorocentrum texanum* Henrichs, Steidinger, P.S.Scott & L.Campbell, 2013, *Prorocentrum micans* Ehrenberg, 1834 and *Prorocentrum mexicanum* Osorio-Tafall, 1942), *Scrippsiella*/*Peridinium* (*Scrippsiella hangoei* (J.Schiller) J.Larsen, 1995 and *Peridinium aciculiferum* Lemmermann, 1900) and *Scrippsiella*/*Theleodinium* (*Scrippsiella acuminata* and *Theleodinium calcisporum* Craveiro, Pandeirada, Daugbjerg, Moestrup & Calado, 2013) species, it could not be established in view of the inadequate set of recognisable identifiers in the target region. The 18S rRNA region is not useful for differentiating some species because of a low variability (Henrichs et al. 2013­). Irrefutably, incorporating different target regions leads to improved detection efficiency (Smith et al. 2017, Sildever et al. 2019, Atherton and Jondelius 2020). Indeed, the reference database deficiency may also hinder the correct interpretation of metabarcoding data (some sequences were assigned to references deposited as sp.: for example *Fragilidium* sp., *Gymnodinium* sp., *Proterythropsis* sp., *Scrippsiella* sp. and *Warnowia* sp.). A practical solution to such an issue might be achieved through filling the taxonomic gaps with integrated registration for both morphological and genetic information. The development of reliable databases with data from different regions will further facilitate the precise identification of OTUs. In the current study, the morphological dataset covered 13 *Protoperidinium* species (nine identified at the species level and four at the genus level), whereas the filtered NGS dataset, including only sequence with similarity ≥ 98% with the reference was related to only one species. Actually, there were more *Protoperidinium* species determined in the molecular data, but were excluded from any further analyses due to their low similarity (below the set threshold).

Given the fact that the application of different methods increases the potential for substantial growth in biodiversity studies, both approaches can benefit from the integration of the information contained in the datasets generated by metabarcoding and morphology methods (Zimmermann et al. 2015). Our results highlight the significance of the combined data for a better interpretation of the results and advanced understanding of the current species diversity. On one hand, environmental DNA metabarcoding, shedding new light on the species richness and infinite variety, could speed up the proper identification of dinoflagellate resting stages, refine their cryptic and pseudo-cryptic alignment, providing a vivid picture of the immense benthic microalgal diversity. For example, five different species were genetically identified within genus *Alexandrium*, though, discerned under a light microscopy, there were two species and seven noted as sp. On the other hand, the traditional identification technique via LM proved to be even more indispensable for the correct validation of the data obtained by metabarcoding. Yet, some identified morphospecies exist that could not be detected using the NGS analyses (eight *Protoperidinium* species were precisely morphologically identified as full cysts vs. only one species determined genetically).

The combined results broaden our knowledge on the dinoflagellate diversity in Black Sea sediments and highlight the importance of carrying out integrated investigations. Moreover, the presence of species, both typical and exceptional for the present-day plankton community, defines the role of sediments as seed banks where resting stages accumulate over time. Some cysts are ubiquitously distributed in Black Sea sediments, whereas others are irregularly scattered in patchy dispersion. Most notably, however, is that, unlike other studies (Gong et al. 2015), a separate depth-dependent gradient of dinoflagellate cyst diversity was not found. In a nutshell, the patterns of cysts richness, quantitative and spatial distribution should be more thoroughly explored within the context of environmental conditions, which might additionally support the overall analysis of the results. Further complex multidisciplinary studies are needed to unravel the factors underlying the structure of plankton cyst banks in the sediments.

## Supplementary Material

12D7AE79-559E-5FAF-BA07-DD9875118836 10.3897/BDJ.8.e55172.suppl1 Supplementary material 1 Minimum and maximum abundance of the dinoflagellate species identified in the Black Sea sediment samples by morphological and molecular approach Data type Table File: oo_433752.DOCX https://binary.pensoft.net/file/433752 Dzhembekova, N., Rubino, F., Nagai, S., Zlateva, I., Slabakova, N., Ivanova, P., Slabakova, V., Moncheva, S.

## Figures and Tables

**Table 1.** Overview of samplings in the Black Sea

**Date of sampling**	**Sampling station**	**Latitude N /Longitude E**	**Station depth**	**Sampling device**
17.5.2016	1	46°12,098'/30°49,649′	28.6	multicorer
18.5.2016	2	45°30,969′/29°51,728'	20.4	multicorer
19.5.2016	3	45°11,999′/29°48,616'	20.5	multicorer
19.5.2016	4	45°18,676'/29°51,200′	22.0	multicorer
21.5.2016	5	46°30,535′/30°49,432′	13.1	Van-Veen grab
21.5.2016	6	46°19,474'/31°27,999′	16.0	multicorer
24.5.2016	7	43°22,049'/31°49,994′	1933.0	multicorer
26.5.2016	8	42°14,070′/39°53,161'	1904.0	multicorer
29.5.2016	9	42°07,359′/41°36,987'	37.0	Van-Veen grab
30.5.2016	10	41°54,259′/41°44,948′	42.0	Van-Veen grab
30.5.2016	11	41°45,763′/41°42,883′	63.0	Van-Veen grab
2.6.2016	12	43°31,558′/36°04,183'	2100.0	multicorer
3.6.2016	13	44°38,163′/31°23,298′	391.0	multicorer

**Table 2.** Number of samples with shared species identified by the morphological and NGS approach.

**SHARED SPECIES**	Number of samples (n)		
	Full cysts	Empty cysts	Molecular
*Alexandrium minutum*	4	0	5
*Diplopsalis lenticula*	7	8	1
*Gonyaulax spinifera*	1	0	10
*Gymnodinium litoralis*	4	1	12
*Gymnodinium nolleri*	12	8	13
*Lingulodinium polyedra*	9	9	4
*Pentapharsodinium dalei*	8	3	4
*Pentapharsodinium tyrrhenicum*	10	9	13
*Polykrikos hartmannii*	3	0	9
*Protoceratium reticulatum*	2	0	10

**Table 3.** Possible biases explaining discrepancies between morphological and NGS data in sediment resting cyst analyses (modified from Harvey et al., 2017).

**Bias type**	**Name**	**Description**	**Possible effect**
Sampling	Fractional sampling	Morphological and NGS methods were applied to a subset of each sample	Numbers of individuals and possibly taxonomic composition may not be identical between subsamples
	DNA extraction effectiveness	DNA extraction from sediment samples is difficult/ challenging	Taxa and/or abundances may be under-represented in the resulting data
Organism	Life stage identifications	Sequencing technologies cannot differentiate between different phytoplankton life stages (cysts/vegetative cells)	DNA from sedimented vegetative cells and/or the extracellular DNA preserved in the sediment may affect the reliability of the results
PCR	Taxonomic variation	Target gene copy number vary amongst taxa	Taxa with more target gene copies per cell may be favoured over those with fewer copies
	Primer bias	Primer design supports some taxa against others	Favoured taxa may be preferentially amplified, whereas other taxa may be missed
	Amplification bias	DNA from some taxa is more easily amplified than from other taxa	Privileged taxa may be preferentially amplified and over-represented
Database	Database composition	Morphological knowledge and/or NGS databases may not include all sampled taxa	Inability to assign taxonomy to organisms and/or incorrect assignments are made
	Data resolution	Available morphological and DNA sequence data resolution may vary amongst taxa	Morphology and/or NGS data may not contain sufficient information to assign taxonomy at finer scales (e.g. genus, species)
	Database errors	Possible mismatch of taxonomic information to morphological or DNA sequence data	Incorrect taxonomic assignments
