# Supplementary material for: Comparative analysis of morphological and molecular approaches integrated into the study of the dinoflagellate biodiversity within the recently deposited Black Sea sediments – benefits and drawbacks
Source: Biodivers Data J. 2020 Aug 18;8:e55172. doi: 10.3897/BDJ.8.e55172 (PMC7447646; doi:10.3897/BDJ.8.e55172)
Supplement: Supplementary material 1 — Minimum and maximum abundance of the dinoflagellate species identified in the Black Sea sediment samples by morphological and molecular approach [file bdj-08-e55172-s001.docx]

**Supplementary Table 1.** Minimum and maximum abundance of the dinoflagellate species identified in the Black Sea sediment samples by morphological and molecular approach

| ***Species*** | | | **Morphology full cysts** | | | **Morphology empty cysts** | | | | **Molecular** | | |
| --- | --- | --- | --- | --- | --- | --- | --- | --- | --- | --- | --- | --- |
|  |  |  | *sf** | *min* | *max* | *sf** | | *min* | *max* | *sf** | *min* | *max* |
|  |  |  | (cysts g-1 Dws*) | | | (cysts g-1 Dws*) | | | | (MPSs*) | | |
| *Akashiwo sanguinea* (K.Hirasaka) Gert Hansen & Moestrup, 2000 | | |  | 0 | 0 |  | | 0 | 0 |  | 2 | 39 |
| *Alexandrium affine* (H.Inoue & Y.Fukuyo) Balech, 1995 | | |  | 0 | 0 |  |  | 0 | 0 | ***14*** | n/a | n/a |
| *Alexandrium andersonii* Balech, 1990 | | |  | 0 | 0 |  |  | 0 | 0 | ***52*** | n/a | n/a |
| *Alexandrium margalefii* Balech, 1994 | | | ***26*** | n/a | n/a |  |  | 0 | 0 |  | 0 | 0 |
| *Alexandrium minutum* Halim, 1960 | | |  | 9 | 39 |  |  | 0 | 0 |  | 4 | 276 |
| *Alexandrium pacificum* R.W.Litaker, 2014 | | |  | 0 | 0 |  | | 0 | 0 | ***17*** | n/a | n/a |
| *Alexandrium* sp.1 | | |  | 2 | 19 |  | | 0 | 0 |  | 0 | 0 |
| *Alexandrium* sp.10 | | |  | 10 | 19 |  |  | 0 | 0 |  | 0 | 0 |
| *Alexandrium* sp.11 | | |  | 0 | 0 | ***17*** | | n/a | n/a |  | 0 | 0 |
| *Alexandrium* sp.4 | | |  | 2 | 167 |  | | 2 | 6 |  | 0 | 0 |
| *Alexandrium* sp.6 | | | ***37*** | n/a | n/a |  |  | 0 | 0 |  | 0 | 0 |
| *Alexandrium* sp.7 | | |  | 2 | 68 |  |  | 0 | 0 |  | 0 | 0 |
| *Alexandrium* sp.8 | | | ***9*** | n/a | n/a |  |  | 0 | 0 |  | 0 | 0 |
| *Alexandrium tamutum* M.Montresor, A.Beran & U.John, 2004 | | |  | 0 | 0 |  |  | 0 | 0 |  | 9 | 87 |
| *Amphidinium carterae* Hulburt, 1957 | | |  | 0 | 0 |  |  | 0 | 0 | ***2*** | n/a | n/a |
| *Amphidoma languida* Tillmann, Salas & Elbrachter, 2012 | | |  | 0 | 0 |  |  | 0 | 0 |  | 1 | 429 |
| *Ansanella granifera* H.J.Jeong, S.H.Jang, Moestrup & N.S.Kang, 2014 | | |  | 0 | 0 |  |  | 0 | 0 |  | 6 | 290 |
| *Azadinium dalianense* Zhaohe Luo, Haifeng Gu & Tillmann, 2013 | | |  | 0 | 0 |  |  | 0 | 0 | ***15*** | n/a | n/a |
| *Azadinium dexteroporum* I.Percopo & A.Zingone, 2013 | | |  | 0 | 0 |  |  | 0 | 0 |  | 2 | 4 |
| *Azadinium polongum* Tillmann, 2012 | | |  | 0 | 0 |  |  | 0 | 0 |  | 9 | 709 |
| *Azadinium poporum* Tillmann & Elbrächter, 2011 | | |  | 0 | 0 |  |  | 0 | 0 |  | 4 | 6 |
| *Azadinium trinitatum* Tillmann & Nézan 2014 | | |  | 0 | 0 |  |  | 0 | 0 | ***41*** | n/a | n/a |
| *Biecheleria* sp. | | |  | 0 | 0 |  |  | 0 | 0 |  | 63 | 59198 |
| *Biecheleriopsis adriatica* Moestrup, Lindberg & Daugbjerg, 2009 | | |  | 0 | 0 |  |  | 0 | 0 |  | 6 | 2059 |
| *Brandtodinium nutricula* (Brandt) Probert & Siano, 2014 | | |  | 0 | 0 |  |  | 0 | 0 |  | 6 | 74 |
| *Calciodinellum albatrosianum* (Kamptner) Janofske & Karwath, 2000 | | |  | 3 | 778 |  |  | 2 | 2500 |  | 0 | 0 |
| *Calciodinellum operosum* Deflandre, 1947 † | | |  | 9 | 167 |  |  | 3 | 130 |  | 0 | 0 |
| *Calciperidinium asymmetricum* Versteegh, 1993 † | | |  | 3 | 83 | ***117*** | | n/a | n/a |  | 0 | 0 |
| *Ceratium tenue* Ostenfeld & Schmidt, 1901 | | |  | 0 | 0 |  | | 0 | 0 | ***4*** | n/a | n/a |
| *Ceratocorys horrida* Stein, 1883 | | |  | 0 | 0 |  |  | 0 | 0 | ***2*** | n/a | n/a |
| *Diplopelta parva* (T.H.Abé) K.Matsuoka, 1988 | | | ***37*** | n/a | n/a |  |  | 0 | 0 |  | 0 | 0 |
| *Diplopsalis lenticula* Bergh, 1881 | | |  | 3 | 53 |  |  | 2 | 111 | ***25*** | n/a | n/a |
| *Dissodinium pseudocalani* (Gonnert) Drebes ex Elbrachter & Drebes, 1978 | | |  | 6 | 29 |  |  | 0 | 0 |  | 0 | 0 |
| *Dissodinium pseudolunula* Swift ex Elbrächter & Drebes, 1978 | | |  | 0 | 0 |  |  | 0 | 0 |  | 1 | 122 |
| *Ensiculifera carinata* Matsuoka, Kobayashi & Gains, 1990 | | |  | 2 | 271 |  |  | 0 | 0 |  | 0 | 0 |
| *Euduboscquella cachoni* (Coast) D.W.Coast, T.R.Bachvaroff & C.F.Delwiche, 2012 | | |  | 0 | 0 |  |  | 0 | 0 | ***8*** | n/a | n/a |
| *Follisdinellum splendidum* Versteegh, 1993 † | | |  | 2 | 3 | ***5*** | | n/a | n/a |  | 0 | 0 |
| *Fragilidium duplocampanaeforme* Nézan & Chomérat, 2009 | | |  | 0 | 0 |  | | 0 | 0 | ***21*** | n/a | n/a |
| *Fragilidium* sp. | | |  | 0 | 0 |  |  | 0 | 0 | ***5*** | n/a | n/a |
| *Gonyaulax cochlea* Meunier, 1919 | | |  | 0 | 0 |  |  | 0 | 0 |  | 7 | 52 |
| *Gonyaulax* sp. | | |  | 2 | 111 |  |  | 2 | 56 |  | 0 | 0 |
| *Gonyaulax spinifera* (Claparède & Lachmann) Diesing, 1866 | | | ***3*** | n/a | n/a |  |  | 0 | 0 |  | 2 | 3672 |
| *Gymnodinium aureolum* (E.M.Hulburt) Gert Hansen, 2000 | | |  | 0 | 0 |  |  | 0 | 0 |  | 38 | 9983 |
| *Gymnodinium catenatum* H.W.Graham, 1943 | | |  | 0 | 0 |  |  | 0 | 0 |  | 805 | 35999 |
| *Gymnodinium dorsalisulcum* (Hulbert, J.J.A.McLaughlin & Zahl) Shauna Murray, Salas & Hallegraeff, 2007 | | |  | 0 | 0 |  |  | 0 | 0 |  | 2 | 244 |
| *Gymnodinium impudicum* (S.Fraga & I.Bravo) Gert Hansen & Moestrup, 2000 | | |  | 9 | 19 |  |  | 0 | 0 |  | 0 | 0 |
| *Gymnodinium litoralis* A.ReÃ±é, 2011 | | |  | 3 | 25 | ***33*** | | n/a | n/a |  | 1 | 25 |
| *Gymnodinium nolleri* M.Ellegaard & Ø.Moestrup, 1999 | | |  | 2 | 56 |  | | 3 | 167 |  | 14 | 2686 |
| *Gymnodinium* sp. GSSW10 | | |  | 0 | 0 |  |  | 0 | 0 |  | 121 | 2622 |
| *Gymnodinium* sp. NVA/RUS/2008 | | |  | 0 | 0 |  |  | 0 | 0 |  | 3 | 220 |
| *Gymnodinium* sp.5 | | | ***37*** | n/a | n/a |  |  | 0 | 0 |  | 0 | 0 |
| *Gymnodinium uncatenatum* (Hulburt) Hallegraeff, 2002 | | | ***15*** | n/a | n/a |  |  | 0 | 0 |  | 0 | 0 |
| *Gyrodinium spirale* (Bergh) Kofoid & Swezy, 1921 | | |  | 0 | 0 |  |  | 0 | 0 | ***2*** | n/a | n/a |
| *Heterocapsa niei* (Loeblich III) Morrill & Loeblich III, 1981 | | |  | 0 | 0 |  |  | 0 | 0 |  | 1 | 12 |
| *Heterocapsa rotundata* (Lohmann) Gert Hansen, 1995 | | |  | 0 | 0 |  |  | 0 | 0 |  | 2 | 1059 |
| *Heterocapsa triquetra* (Ehrenberg) F.Stein, 1883 | | |  | 0 | 0 |  |  | 0 | 0 |  | 2 | 19 |
| *Karenia papilionacea* A.J.Haywood & K.A.Steidinger, 2004 | | |  | 0 | 0 |  |  | 0 | 0 |  | 2 | 2389 |
| *Karlodinium veneficum* (D.Ballantine) J.Larsen, 2000 | | |  | 0 | 0 |  |  | 0 | 0 |  | 74 | 3151 |
| *Kryptoperidinium foliaceum* (F.Stein) Lindemann, 1924 | | |  | 9 | 56 |  |  | 0 | 0 |  | 0 | 0 |
| *Lepidodinium* sp. | | |  | 0 | 0 |  |  | 0 | 0 |  | 1 | 971 |
| *Lepidodinium viride* M.Watanabe, S.Suda, I.Inouye, T.Sawaguchi & M.Chihara, 1990 | | |  | 0 | 0 |  |  | 0 | 0 |  | 1 | 416 |
| *Lingulodinium polyedra* (F.Stein) J.D.Dodge, 1989 | | |  | 2 | 1722 |  |  | 3 | 167 |  | 2 | 5 |
| *Margalefidinium polykrikoides*(Margalef) F.Gómez, Richlen & D.M.Anderson, 2017 | | |  | 0 | 0 |  |  | 0 | 0 |  | 2 | 100 |
| *Melodomuncula berlinensis* Versteegh, 1993 † | | |  | 26 | 150 | ***67*** | | n/a | n/a |  | 0 | 0 |
| *Nematodinium armatum* (Dogiel) Kofoid & Swezy, 1921 | | | ***2*** | n/a | n/a |  | | 2 | 100 |  | 0 | 0 |
| *Noctiluca scintillans* (Macartney) Kofoid & Swezy, 1921 | | |  | 0 | 0 |  |  | 0 | 0 |  | 1 | 367 |
| *Oblea rotunda* (Lebour) Balech ex Sournia, 1973 | | |  | 3 | 17 |  |  | 0 | 0 |  | 0 | 0 |
| *Paragymnodinium shiwhaense* N.S.Kang, H.J.Jeong, Moestrup & W.Shin, 2011 | | |  | 0 | 0 |  |  | 0 | 0 |  | 1 | 63 |
| *Paulsenella vonstoschii* Drebes & Schnepf | | |  | 0 | 0 |  |  | 0 | 0 |  | 9 | 49 |
| *Pelagodinium bei* (H.J.Spero) Siano, Montresor, Probert & Vargas, 2010 | | |  | 0 | 0 |  |  | 0 | 0 |  | 3 | 899 |
| *Pentapharsodinium dalei* Indelicato & Loeblich III, 1986 | | |  | 4 | 130 |  |  | 19 | 19 |  | 1 | 12 |
| *Pentapharsodinium tyrrhenicum* (Balech) Montresor, Zingone & Marino, 1993 | | |  | 2 | 275 |  |  | 2 | 225 |  | 4 | 1583 |
| *Peridiniopsis penardii* (Lemmermann) Bourrelly, 1968 | | |  | 0 | 0 |  |  | 0 | 0 |  | 4 | 5 |
| *Peridiniopsis polonicum* (Woloszynska) Bourrelly, 1968 | | |  | 0 | 0 |  |  | 0 | 0 | ***4*** | n/a | n/a |
| *Peridinium aciculiferum* Lemmermann, 1900 | | |  | 0 | 0 |  |  | 0 | 0 |  | 1 | 18 |
| *Polarella glacialis* M.Montresor, G.Procaccini & D.K.Stoecker, 1999 | | |  | 0 | 0 |  |  | 0 | 0 |  | 1 | 44 |
| *Polykrikos geminatus* (Schütt) D.Qiu & S.Lin, 2013 | | |  | 0 | 0 |  |  | 0 | 0 |  | 53 | 1042 |
| *Polykrikos hartmannii* W.M.Zimmermann, 1930 | | |  | 2 | 9 |  |  | 0 | 0 |  | 1 | 326 |
| *Polykrikos schwartzii* Bütschli, 1873 | | | ***2*** | n/a | n/a |  |  | 5 | 19 |  | 0 | 0 |
| *Posoniella tricarinelloides* (G.Versteegh) Streng, Banasová, D.Reháková & H.Willems | | |  | 3 | 333 | ***33*** | | n/a | n/a |  | 0 | 0 |
| *Prorocentrum cordatum* (Ostenfeld) J.D.Dodge, 1975 | | |  | 0 | 0 |  | | 0 | 0 |  | 10 | 538 |
| *Prorocentrum* sp. | | |  | 0 | 0 |  |  | 0 | 0 |  | 13 | 6120 |
| *Proterythropsis* sp. | | |  | 0 | 0 |  |  | 0 | 0 |  | 1 | 106 |
| *Protoceratium reticulatum* (Claparède & Lachmann) Bütschli, 1885 | | |  | 2 | 3 |  |  | 0 | 0 |  | 9 | 302 |
| *Protodinium simplex* Lohmann, 1908 | | |  | 0 | 0 |  |  | 0 | 0 |  | 4 | 4383 |
| *Protoperidinium claudicans* (Paulsen, 1907) Balech, 1974 | | |  | 0 | 0 |  |  | 2 | 50 |  | 0 | 0 |
| *Protoperidinium compressum* (Abé) Balech, 1974 | | | ***2*** | n/a | n/a |  |  | 9 | 56 |  | 0 | 0 |
| *Protoperidinium conicum* (Gran) Balech, 1974 | | |  | 9 | 50 | ***50*** | | n/a | n/a |  | 0 | 0 |
| *Protoperidinium excentricum* (Paulsen, 1907) Balech, 1974 | | |  | 0 | 0 |  | | 0 | 0 | ***3*** | n/a | n/a |
| *Protoperidinium oblongum* (Aurivillius) Parke & Dodge, 1976 | | | ***9*** | n/a | n/a |  |  | 0 | 0 |  | 0 | 0 |
| *Protoperidinium parthenopes* A.Zingone & M.Montresor, 1988 | | |  | 2 | 19 | ***37*** | | n/a | n/a |  | 0 | 0 |
| *Protoperidinium* sp.1 | | |  | 2 | 97 |  | | 2 | 74 |  | 0 | 0 |
| *Protoperidinium* sp.4 | | |  | 0 | 0 | ***2*** | | n/a | n/a |  | 0 | 0 |
| *Protoperidinium* sp.5 | | |  | 9 | 19 |  | | 0 | 0 |  | 0 | 0 |
| *Protoperidinium* sp.6 | | | ***2*** | n/a | n/a | ***2*** | | n/a | n/a |  | 0 | 0 |
| *Protoperidinium steidingerae* Balech, 1979 | | | ***9*** | n/a | n/a |  | | 0 | 0 |  | 0 | 0 |
| *Protoperidinium subinerme* (Paulsen) Loeblich III, 1969 | | |  | 0 | 0 | ***17*** | | n/a | n/a |  | 0 | 0 |
| *Protoperidinium thorianum* (Paulsen, 1905) Balech, 1974 | | | ***2*** | n/a | n/a |  | | 0 | 0 |  | 0 | 0 |
| *Scrippsiella acuminata* (Ehrenberg) Kretschmann, Elbrächter, Zinssmeister, S.Soehner, Kirsch, Kusber & Gottschling, 2015 | | |  | 175 | 4475 |  |  | 31 | 2700 |  | 0 | 0 |
| *Scrippsiella* cf. *erinaceus* (Kamptner) J.Kretschmann, C.Zinssmeister & M.Gottschling, 2014 | | |  | 2 | 93 | ***5*** | | n/a | n/a |  | 0 | 0 |
| *Scrippsiella lachrymosa* J.Lewis, 1991 | | |  | 0 | 0 | ***19*** | | n/a | n/a |  | 0 | 0 |
| *Scrippsiella precaria* M.Montresor & A.Zingone, 1988 | | |  | 0 | 0 |  | | 0 | 0 |  | 1 | 121 |
| *Scrippsiella ramonii* M.Montresor, 1995 | | | ***5*** | n/a | n/a |  |  | 0 | 0 |  | 0 | 0 |
| *Scrippsiella* sp. HZ-2005 | | |  | 0 | 0 |  |  | 0 | 0 |  | 1 | 722 |
| *Scrippsiella* sp. SCKS 0701 | | |  | 0 | 0 |  |  | 0 | 0 |  | 2 | 4885 |
| *Scrippsiella* sp.1 | | |  | 37 | 1463 |  |  | 19 | 750 |  | 0 | 0 |
| *Scrippsiella* sp.4 | | |  | 6 | 37 | ***2*** | | n/a | n/a |  | 0 | 0 |
| *Scrippsiella* sp.5 | | |  | 2 | 128 | ***19*** | | n/a | n/a |  | 0 | 0 |
| *Scrippsiella* sp.6 | | |  | 5 | 28 |  | | 2 | 56 |  | 0 | 0 |
| *Scrippsiella trifida* J.Lewis, 1991 | | |  | 5 | 241 |  |  | 17 | 150 |  | 0 | 0 |
| *Warnowia* sp. BSL-2009a | | |  | 0 | 0 |  |  | 0 | 0 |  | 1 | 78 |
| *Woloszynskia pascheri* (Suchlandt) Stosch, 1973 | | |  | 0 | 0 |  |  | 0 | 0 |  | 6 | 2758 |
| **sf - single frequencies*  **Dws - dry wet sediment*  **MPSs - massive parallel sequences* | | |  |  |  |  |  |  |  |  |  |  |
| LEGEND: | |  | | | | | |  |  |  |  |  |
| *Morphology full cysts data set:* | | | | | | | |  |  |  |  |  |
|  | | not represented in the samples | | | | | |  |  |  |  |  |
|  | | top 10 species with highest abundance per sample | | | | | |  |  |  |  |  |
|  | | species with lowest abundance per sample | | | | | |  |  |  |  |  |
|  | | lowest single frequencies | | | | | |  |  |  |  |  |
|  | | highest single frequencies | | | | | |  |  |  |  |  |
| *Morphology empty cysts data set:* | | | | | | | |  |  |  |  |  |
|  | | not represented in the samples | | | | | |  |  |  |  |  |
|  | | top 10 species with highest abundance per sample | | | | | |  |  |  |  |  |
|  | | species with lowest abundance per sample | | | | | |  |  |  |  |  |
|  | | lowest single frequencies | | | | | |  |  |  |  |  |
|  | | highest single frequencies | | | | | |  |  |  |  |  |
| *Molecular data set:* | | | | | | | |  |  |  |  |  |
|  | | not represented in the samples | | | | | |  |  |  |  |  |
|  | | top 10 species with highest abundance per sample | | | | | |  |  |  |  |  |
|  | | species with lowest abundance per sample | | | | | |  |  |  |  |  |
|  | | lowest single frequencies | | | | | |  |  |  |  |  |
|  | | highest single frequencies | | | | | |  |  |  |  |  |
